# The Different Pathways of Epicardial Adipose Tissue across the Heart Failure Phenotypes: From Pathophysiology to Therapeutic Target

**DOI:** 10.3390/ijms24076838

**Published:** 2023-04-06

**Authors:** Valentina A. Rossi, Martin Gruebler, Luca Monzo, Alessandro Galluzzo, Matteo Beltrami

**Affiliations:** 1 University Heart Center, Department of Cardiology, University Hospital of Zurich, 8091 Zurich, Switzerland; 2 Regional Hospital Neustadt, 2700 Wiener Neustadt, Austria; 3 Faculty of Medicine, Medical University of Graz, 8036 Graz, Austria; 4 Faculty of Medicine, Sigmund Freud University Vienna, 1020 Vienna, Austria; 5 Centre d’Investigations Cliniques Plurithématique 1433 and Inserm U1116, Université de Lorraine, CHRU Nancy, FCRIN INI-CRCT (Cardiovascular and Renal Clinical Trialists), 54035 Nancy, France; 6 Cardiology Unit, Santa Croce Hospital, Moncalieri, 10024 Turin, Italy; 7 Azienda USL Toscana Centro, Cardiology Unit, San Giovanni di Dio Hospital, 50143 Florence, Italy; beltrami.matteo1@gmail.com

**Keywords:** epicardial adipose tissue, biomolecular pathways, heart failure, cardiovascular disease

## Abstract

Epicardial adipose tissue (EAT) is an endocrine and paracrine organ constituted by a layer of adipose tissue directly located between the myocardium and visceral pericardium. Under physiological conditions, EAT exerts protective effects of brown-like fat characteristics, metabolizing excess fatty acids, and secreting anti-inflammatory and anti-fibrotic cytokines. In certain pathological conditions, EAT acquires a proatherogenic transcriptional profile resulting in increased synthesis of biologically active adipocytokines with proinflammatory properties, promoting oxidative stress, and finally causing endothelial damage. The role of EAT in heart failure (HF) has been mainly limited to HF with preserved ejection fraction (HFpEF) and related to the HFpEF obese phenotype. In HFpEF, EAT seems to acquire a proinflammatory profile and higher EAT values have been related to worse outcomes. Less data are available about the role of EAT in HF with reduced ejection fraction (HFrEF). Conversely, in HFrEF, EAT seems to play a nutritive role and lower values may correspond to the expression of a catabolic, adverse phenotype. As of now, there is evidence that the beneficial systemic cardiovascular effects of sodium-glucose cotransporter-2 receptors-inhibitors (SGLT2-i) might be partially mediated by inducing favorable modifications on EAT. As such, EAT may represent a promising target organ for the development of new drugs to improve cardiovascular prognosis. Thus, an approach based on detailed phenotyping of cardiac structural alterations and distinctive biomolecular pathways may change the current scenario, leading towards a precision medicine model with specific therapeutic targets considering different individual profiles. The aim of this review is to summarize the current knowledge about the biomolecular pathway of EAT in HF across the whole spectrum of ejection fraction, and to describe the potential of EAT as a therapeutic target in HF.

## 1. Introduction

Epicardial adipose tissue (EAT) is a peculiar fat depot located between the myocardium and the visceral layer of the epicardium. Interestingly, EAT evolved from brown adipose tissue and shares the same embryologic origin as omental and mesenteric fat [1].

EAT works as a lipid-storing depot, but it has been also identified as an endocrine organ secreting hormones, part of the immune signalling network, and as an inflammatory tissue secreting cytokines and chemokines [2]. Notably, due to its close proximity and shared microvascular network, the EAT-myocardium microenvironment can be a place of intensive exchange of paracrine regulators or metabolic substrates, and it may serve as a transducer of systemic metabolic state, inflammation, immunologic response, or neurohumoral activation on the cardiac muscle [3,4]. In normal conditions, EAT plays several physiologic roles. Firstly, it works as a mechanical protectant for the surrounding coronary arteries, buffering them against the torsion induced by the arterial pulse wave and cardiac contraction [5]. Furthermore, it has a fundamental thermogenic function, serving as a protector against myocardial hypothermia [6]. EAT also houses a wide range of immune cells, mostly M2-like macrophages (with a 4:1 M2:M1 ratio), but also eosinophils and regulatory T cells which secrete interleukin (IL)-4/IL-13 and IL-10, respectively, thus polarizing macrophages towards an anti-inflammatory phenotype [7]. Finally, EAT plays a key role in the myocardial energy metabolism. Importantly, free fatty acids (FFAs) oxidation accounts for about 50–70% of the myocardial energy production, representing the most important energy source in a healthy heart [8]. As such, epicardial fat has been proposed as a buffer between the myocardium and the local vasculature, possibly protecting the heart against excessively high levels of FFAs [9,10]. Furthermore, the high lipolytic activity of EAT suggests that it may also play a role as a ready source of FFAs to meet increased myocardial energy demand through the β-oxidation and oxidative phosphorylation in mitochondria, and, thus, nourish the healthy myocardium [11].

Furthermore, EAT has been shown to be a highly active biological tissue secreting many adipokines that have the capacity to affect the adjacent myocardium [8]. Among these, adiponectin—an adipocyte-derived cytokine with cardioprotective effects—appears to be most important, particularly due to its anti-atherosclerotic, anti-apoptosis, oxidative stress diminishing, fibrosis-reducing, and anti-congestion properties [12,13]. Several other adipokines have been described to be secreted by EAT, such as interleukin (IL)-1β, -6, -8 and -10, tumor necrosis factor α (TNF-α), monocyte chemo-attractive protein 1 (MCP-1), leptin, and plasminogen activator inhibitor 1 (PAI-1) [14].

### 1.1. The Role of Epicardial Adipose Tissue in Cardiovascular Disease

Systemic inflammation and metabolic disorders cause EAT proliferation and deranged adipogenesis [15]. As EAT expands, it becomes hypoxic and dysfunctional [16], resulting in a shift in its metabolic profile with the activation of stress pathways such as endoplasmic reticulum stress, oxidative stress, and inflammasome [17]. As a consequence, adipose tissue produces several inflammatory mediators (i.e., IL1-β, IL-6; IL-8, IL-10; TGF-β, TNF-α, MCP-1, PAI-I), and induces adiponectin production drops and leptin increases. This last event results in a significant increase in immune cells in the EAT (PMID: 16751422) mediated by their membrane leptin receptor (LEP-R) [18,19]. Upregulation of the immune response signalling and pro-inflammatory state in the EAT might, in ultimate analysis, influence the development of cardiovascular disease, namely coronary artery disease (CAD) [20,21], cardiac arrhythmias [22,23], and heart failure (HF) [24,25]. The EAT of HF patients was characterized by pronounced immune activation, mainly dominated by the accumulation of T lymphocytes. Indeed, in the EAT of HF patients, these immune cells were highly expanded and in particular constituted by clonally expanded IFN-γ^+^ effector memory T lymphocytes [26]. The established local proinflammatory environment is deemed to cause myocardial microvascular dysfunction and fibrosis [15], responsible in turn for the development of ventricular hypertrophy, diastolic dysfunction, conduction abnormalities, and increased cardiac filling pressures [27,28,29,30], all of which are highly prevalent in HF. In this setting, increased EAT thickness may also change myocardial substrate utilization, with an increased reliance on FFAs oxidation for energy and a concomitant impaired oxygen use, contributing to a reduction in cardiac reserve and aerobic capacity [31]. Myocardial metabolic remodeling with a switch from FFAs to energetically more effective substrates, such as ketone bodies, is usually needed to preserve myocardial efficiency [32].

However, the available—although limited—literature suggests that the EAT may exert a different pathophysiological effect in HF in relation to the ejection fraction status. In this review, we summarize the currently available evidence regarding the role of EAT in the development of HF, highlighting the different pathophysiological pathways in HF with reduced (HFrEF), mildly reduced (HFmrEF), and preserved (HFpEF) ejection fraction.

### 1.2. Quantification of Epicardial Adipose Tissue

Transthoracic echocardiography is a simple, cost-effective, and readily available method which allows the measurement of EAT thickness. EAT thickness is measured as the echo-free space between the outer wall of the right ventricle and the visceral pericardium in the parasternal long-axis view at end-systole, perpendicularly to the aortic annulus. This point presents with the highest absolute EAT thickness [10,33]. Another measurement is usually performed between the outer wall of the right ventricle and the visceral pericardium in the parasternal short-axis view at end-systole, perpendicularly to the papillary muscles [1]. In some studies, the echocardiographic measurement of EAT thickness has been shown to have an excellent interobserver and intraobserver agreement, whereas other studies found it to be poor [1,34,35]. The most relevant limitation of measuring EAT by transthoracic echocardiography is the impossibility to provide information about the total fat volume. This issue is overcome by cardiac magnetic resonance imaging (CMR). CMR, despite being more time-consuming and expensive, allows a more reliable measurement of the global cardiac EAT volume and, as such, represents the gold-standard for its assessment [36]. Similarly, cardiac computer tomography (CT) has a good spatial resolution and has a highly reproducible correlation coefficient [37]. Moreover, there was a positive correlation between pericoronary adipose tissue assessed by CT and worse clinical outcome independently of calcium score [38]. A recent paper demonstrates an association between EAT volume, CAD extent, and impaired left ventricle global longitudinal strain [39]. CT-radiomic is a new and promising method based on the extraction of mineable data from CT to better define and characterize EAT, which has been shown to have a prognostic value in patients with atrial fibrillation [40].

## 2. Epicardial Adipose Tissue in Heart Failure with Preserved Ejection Fraction

HFpEF is a heterogenous and incompletely understood syndrome with specific molecular, genetic, and metabolomic features, all of which reflect on vascular and myocardial cell adaptations [41]. HFpEF encompasses different pathophysiological pathways and cardiac structural profiles compared to HFrEF [42]. Therefore, the HF classification criteria are often elusive and mainly based upon ejection fraction rather than on distinct clinical, metabolic, and laboratory phenotypes [43]. Patients affected by metabolic syndrome (i.e., presenting with metabolic alterations such as obesity, diabetes, and arterial hypertension) present with a particular HFpEF phenotype characterized by relatively low levels of natriuretic peptides, impaired renal function, and only modestly increased cardiac volumes [15]. Altered cardiac hemodynamics in obese people with HFpEF are mainly related to impaired ventricular distensibility, which disproportionately increases ventricular filling pressures. There is increasing evidence that obesity induces the dysregulation of the nitric oxide–cyclic guanosine monophosphate–protein kinase G signaling cascade via obesity-induced proinflammatory pathways [44]. Consequently, inflammation leads to endothelial dysfunction, microvascular myocardial alterations, mitochondrial dysfunction, relative hypoxia and, finally, increased myocardial fibrosis [44,45]. In particular, the interplay of cytokines such as TNF-α, IL-1β, IL-10, IL-4, and IL-13 seems to play a crucial role in modulating LV remodeling and myocardial fibrosis and repair in HFpEF [46,47]. EAT may represent the local mediator and promote proinflammatory adverse effects by expressing changes in the inflammasome and developing a proatherogenic transcriptional profile with increased synthesis of biologically active adipokines with proinflammatory properties [48,49]. This may be the case independently of the level of inflammation in other fat depots, supporting the hypothesis of a local, paracrine effect of EAT [17,50]. As such, EAT might promote the secretion of proinflammatory adipokines via deranged adipogenesis and, thus, induce atrial and ventricular fibrosis and alterations of the microcirculation [15]. Additionally, local inflammation and changes in the micro-environment may foster cardiomyocyte dysfunction [15]. This hypothesis is supported by the finding of a direct correlation between intramyocardial adipose tissue, and impaired diastolic function in HFpEF patients, particularly in HFpEF women, regardless of their age, co-morbidities, BMI, and myocardial fibrosis [51]. Furthermore, EAT has been shown to express profibrotic proteins, such as serpine A3, matrix metalloproteinase 14, and inflammatory biomarkers (p53 mRNA), which may work as modulators of HF [48,52].

Higher EAT volumes might negatively affect the left ventricle (LV), impairing its distensibility and leading to the development of HFpEF [53]. In a large meta-analysis investigating 22 studies, a greater EAT was associated with diastolic dysfunction independently of other adiposity measures [28]. In particular, in patients with HFpEF, a positive correlation between increased EAT and a right-sided ventricular constrictive pattern at echocardiography has been observed, thus supporting the hypothesis of a mechanical constrictive effect of EAT on the myocardial distensibility [54]. Similarly, EAT localized near the ventricles has been related to ventricular mass, independently of global measures of adiposity [55].

**Figure 1:**
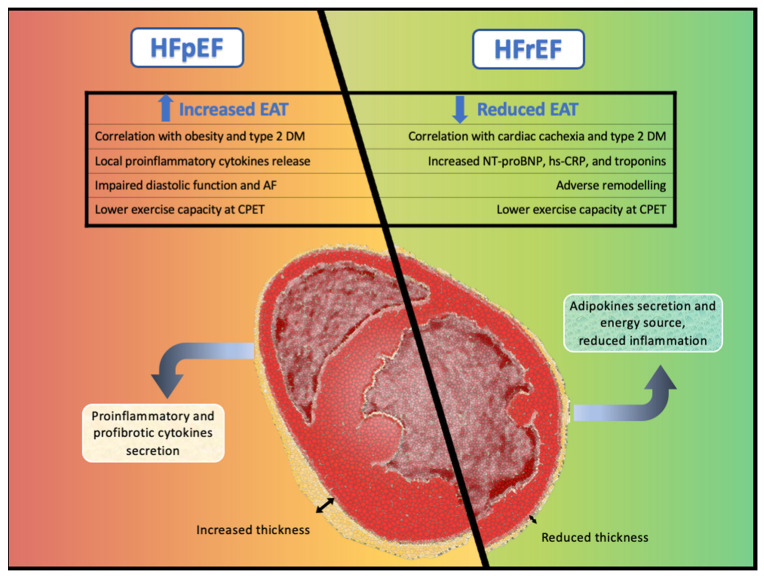
Central illustration. Different pathological pathways and cardiovascular effects of epicardial adipose tissue in heart failure with preserved ejection fraction and in heart failure with reduced ejection fraction.

There is increasing evidence showing that patients with HFpEF have more EAT compared with healthy controls and patients with HFrEF (Figure 1). Moreover, it has been shown that EAT is strongly correlated with increased mortality and new-onset heart failure in both HFpEF and HFmrEF [56,57,58,59]. This association was independent of body mass index (BMI), HF severity, and co-morbidities, thus suggesting a direct role of EAT in the pathophysiology of HFpEF [56,57,58]. Studies investigating the role of EAT in HFpEF are summarized in Table 1.

### 2.1. The Obese HFpEF Phenotype

Obesity is a key component in HFpEF, favoring disease progression, and both increasing myocardial load and worsening HFpEF-related comorbidities, such as arterial hypertension [62]. Lately, research has focused on fat distribution and characteristics in HFpEF, suggesting a particular adverse role for visceral fat and EAT, independently of BMI [48]. Although some trials showed that abdominal adiposity is a risk factor for all-cause mortality, there are increasing data suggesting that EAT may be a better biomarker for overall body fat compared to BMI, particularly in HF [63]. Indeed, pericardial adipose tissue and visceral adipose tissue, but not subcutaneous adipose tissue, have been associated with incident HF and, particularly, with HFpEF [25]. Furthermore, a correlation between EAT thickness and visceral adipose tissue as measured by CT has been observed in HFpEF patients [64]. Interestingly, only epi- and pericardial adipose tissue have been related to a higher mortality risk and with adverse cardiovascular events, also in otherwise apparently healthy individuals [25,65]. Although the studies investigating the effects of EAT in HFpEF patients scarcely explored the overall body fat composition, HFpEF patients with an obese phenotype and more EAT present with a higher relative adverse event rate as compared to obese patients with lower EAT mass [58,62].

The relationship between obesity and HFpEF is complex. Increased pericardial fat may exert a direct compressive effect on the right ventricle, thus preventing its distension [31,54]. Obese HFpEF patients display increased EAT thickness and a concentric ventricular remodeling, which in turn are associated with parameters of greater pericardial restraint and ventricular diastolic dysfunction as compared to the non-obese counterparts [31]. On the other hand, a reverse causality may also be hypothesized: local paracrine alteration appearing during the early stages of HF may promote the proliferation of local epicardial adipose tissue [24].

These findings point towards an obesity-related HFpEF phenotype as a separate form of cardiac failure with specific hemodynamic changes and characteristics, which should be specifically addressed.

### 2.2. EAT and Sex Differences

Clinical studies demonstrated conflicting results regarding the amount of EAT in men compared to women [66,67]. In particular, older women and post-menopausal status showed higher EAT volumes compared to younger women. Interestingly, parameters of LV diastolic dysfunction such as E/e’ were significantly related to increased EAT thickness in women, but not in men. Similar results were found for LV systolic function where S’ reduction was directly correlated with the amount of EAT [68]. Unfortunately, we do not know if the differences are based on overall adiposity and visceral fat distribution, which are likely different between genders [55,56].

### 2.3. EAT and Exercise Capacity

An increased EAT volume has been associated with a markedly reduced exercise capacity (i.e., reduced capacity of peripheral oxygen extraction), independent of BMI in HFpEF, but not in HfrEF [54,56]. Subclinical impaired exercise capacity may then lead to worse body composition and even more accumulation of EAT [69]. The remark of a greater exercise intolerance has been particularly observed in HfpEF patients with an obese phenotype and increased EAT thickness as compared to their counterparts with normal EAT values, likely due to a concomitant increase in ventricular filling pressures and pulmonary pressures [60]. Peripheral limitation to exercise is a pivotal cause of exercise intolerance in HFpEF patients, and EAT accumulation directly leads to central and peripheral microvascular dysfunction, regardless of BMI [56]. These data further support the emerging paradigm that excess EAT might contribute to the pathophysiology of patients with obesity-related HFpEF.

Although the evidence on differences concerning a reduced exercise capacity in HFpEF patients according to EAT volume are not concordant, different testing modalities and experimental setup might account for different results (e.g., supine position during invasive hemodynamic assessment) [10].

### 2.4. EAT and Atrial Fibrillation

The expansion of EAT has been associated with both obesity and type 2 diabetes mellitus, two important risk factors for atrial fibrillation [53]. In these settings, EAT-related proinflammatory mediators may induce microvascular dysfunction, leading to intramyocardial fibrosis and electroanatomical remodeling also in the atria, leading to atrial myopathy [15,53]. Atrial fibrillation often represents the first manifestation of an underlying latent HFpEF characterized by atrial myopathy and increased ventricular filling pressures [53]. An increased EAT predicts the incidence of atrial fibrillation in apparently healthy individuals, independently from its localization above the myocardium [70]; although, atrial EAT has been observed to be more increased in HFpEF patients with atrial fibrillation as compared to those without atrial fibrillation [55].

## 3. Epicardial Adipose Tissue in Heart Failure with Reduced Ejection Fraction

The potential role of EAT in HfrEF has not been clearly understood yet and, up to now, only few studies have been conducted in this setting (Table 2). The majority of observational studies in HfrEF patients examined the distribution of EAT via CMR [36,51,71], whereas only a minority investigated EAT thickness via transthoracic echocardiography [56].

Several observations showed that both EAT mass and EAT thickness are significantly reduced in HFrEF patients as compared to both healthy controls and HFpEF patients [36,56,71]. This is seen regardless of the etiology of the underlying cardiomyopathy, but mostly in the presence of known cardiovascular risk factors such as type 2 diabetes mellitus, hypertension, dyslipidemia, or active smoking status [36].

Nevertheless, other studies reported that EAT mass increases in patients with HFrEF [51,71], but with a reduced EAT mass/LV mass ratio and thinner right ventricular EAT thickness as compared to healthy controls and HFpEF patients [36,56,71]. Accordingly, HFrEF patients also display the lowest intramyocardial fat amount as compared to HFpEF patients and to non-HF patients [51]. These findings of a globally reduced EAT mass in patients with HFrEF may be the expression of a particular cachectic phenotype in HFrEF patients characterized by a pathological catabolic state, where the heart fails in sufficiently increasing EAT volume to meet the metabolic requests of a compensatory increased ventricular mass [3,72]. In support of this hypothesis, a reduced EAT in HFrEF patients has been related to worse cardiac function and with adverse myocardial remodeling—in contrast to the previously reported findings in HFpEF patients [56]. This catabolic effect might be driven by increased natriuretic peptides, which have a strong lipolytic effect mostly in HF patients, and this leads to excessive fatty acid mobilization [73].

Thus, a reduced EAT thickness in HFrEF patients might, thus, be a sign of preclinical cardiac cachexia and its assessment might help in individuating those patients with worse cardiovascular prognosis. However, this conclusion is partially speculative and further studies addressing this question are warranted.

### Different Role of EAT in HFrEF as Compared to HFpEF

Pugliese and colleagues were the first to describe EAT-related differences between HFrEF and HFpEF in terms of cardiometabolic profile, hemodynamics, and cardiovascular outcome [56]. In HFrEF patients, a reduced EAT thickness was associated with increased NT-proBNP values, higher markers of inflammation (hs-CRP), and myocardial damage (troponins), contrarily to what is observed in HFpEF patients [56]. These data further support the hypothesis of a higher inflammatory status in HFrEF patients with reduced EAT thickness as a direct consequence of cardiac cachexia and catabolic-related adverse effects [3]. Accordingly, in opposition to the previously reported findings in HFpEF patients, HFrEF patients with a reduced EAT thickness had a worse peripheral muscular capacity of oxygen extraction, and presented with cardiopulmonary exercise intolerance as measured by oxygen consumption (peak VO_2_), regardless of BMI [56]. Increased EAT thickness was associated with better LV global longitudinal strain and left atrial (LA) reservoir function in patients with HFrEF/HFmrEF, but not in patients with HFpEF. Accordingly, EAT thickness > 10 mm was associated with LA dysfunction in HFpEF, but not in HFrEF/HFmrEF [61]. In addition, a decreased EAT in HFrEF has been related with worse cardiovascular outcomes (HF hospitalizations and cardiovascular deaths) after a 21-month follow-up [56].

## 4. Epicardial Adipose Tissue in Heart Failure with Mildly Reduced Ejection Fraction

Although almost all of the available studies investigated HF patients with a LVEF >40% as a single entity [55,57,58], HFmrEF likely represents a milder phenotype of HFrEF rather than a separate entity [74]. To the best of our knowledge, only one study investigated the amount of pericardial fat (defined as the sum of epicardial and paracardial fat) by CT differentiating HFpEF from HFmrEF [24]. In this large, community-based, prospective cohort study, pericardial fat was strongly associated with an increased risk of HFpEF, but only modestly with HFmrEF and lacking in HFrEF [24]. Interestingly, only a mild association with HFmrEF was observed, whereas no association with HFrEF was found [24]. Although only hypothesis-generating, these data point towards a distinguished phenotype, and further studies specifically focusing on HFmrEF are warranted.

## 5. Potential of Epicardial Adipose Tissue in Heart Failure as Therapeutic Target

The role of inflammation in HF pathophysiology has been increasingly acknowledged [75].

In this point of view, EAT might play a key role as pathophysiological mediator in the development of HF. In fact, in pathological conditions, EAT acquires a proatherogenic transcriptional profile resulting in the increased synthesis of biologically active adipocytokines with proinflammatory properties, promoting oxidative stress, and finally causing endothelial damage [76]. As previously stated, the EAT amount is increased in HFpEF as compared to HFrEF. This observation might be of particular interest, since therapeutic efforts for disease-modifying drugs in HFpEF may concentrate on reducing the mass and inflammatory state of EAT [77]. Up to now, two anti-inflammatory drugs, i.e., statins and anticytokine agents, have been specifically investigated for their possible effects on adipose tissue inflammation. Statins have known pleiotropic anti-inflammatory properties in patients with systemic inflammatory disorders such as rheumatoid arthritis [78]. First, experimental data in animal HF models demonstrated that the use of rosuvastatin in addition to standard HF therapy resulted in a significant improvement in cardiac remodeling, which has been likely linked to a reduced myocardial inflammation independently from plasma lipid levels [79]. In line, a robust association between statin therapy and both a reduced EAT thickness and lower levels of EAT-secreted inflammatory mediators has been demonstrated [80]. In a large metanalysis of 17 randomized-controlled clinical trials involving patients with dyslipidemia, statins reduced the incidence of HF-related adverse events regardless of the presence of a previous myocardial infarction, possibly indicating a beneficial effect in HFpEF prevention [81]. Conversely, the same positive effects on morbidity and mortality have not been found in patients with HFrEF [82]. Anticytokine drugs, particularly agents against tumor necrosis factor-α and interleukin 1-β, showed a beneficial effect on hypertension-induced cardiac damage in animal models [83]. The effect of the anti-interleukin-1 agent anakinra on systemic inflammation has been studied in HFpEF patients, producing contradictory results [84,85]. In a large randomized controlled trial, canakinumab has been demonstrated to reduce proinflammatory biomarkers as well as the risk of HF hospitalization in patients with HFpEF [86]. However, this study did not investigate separately patients with HFrEF and HFpEF, and included subjects who suffered from a myocardial infarction and had high parameters of systemic inflammation [86]. Thus, the provided data about canakinumab should be considered as exploratory and hypothesis-generating [86]. On the other side, no beneficial effects have been found for tumor necrosis factor-α antagonists in patients with HFrEF, where their use has been linked to an increased risk of adverse outcomes, likely related to sodium retention secondary to increased aldosterone synthesis [87,88].

Inhibitors of sodium-glucose cotransporter-2 receptors (SGLT-2i) represent a pillar of the modern HF therapy due to their beneficial effect on the reduction of adverse cardiovascular events in HF patients across the entire ejection fraction spectrum, and their broad eligibility [89,90,91]. Although their mechanisms of action in HF are still debated, there is increasing evidence that SGLT-2i can reduce cardiac fibrosis and hypertrophy, lowering LV filling pressures in experimental models of HFpEF [92,93]. In diabetic patients with coronary artery disease, dapagliflozin therapy has been demonstrated to reduce EAT mass and inflammatory parameters such as tumor necrosis factor-α and plasminogen activator inhibitor-1 over a period of 6 months [94]. Similar positive effects of SGLT-2i have been reproduced also in non-diabetic patients with HFrEF. Indeed, in the setting of the EMPA-TROPISM study, empagliflozin has been associated with a significant reduction in EAT volume as measured by cardiac magnetic resonance [95]. Interestingly, this reduction in EAT was accompanied by a reduction in myocardial fibrosis and inflammatory biomarkers related to endothelial dysfunction, leukocyte circulation, and cell adhesion molecules, such as E-selectin [95].

Besides the above-mentioned therapies, metformin and glucagon-like peptide-1 (GLP-1) receptor agonists have also been found to reduce EAT inflammation and EAT mass, respectively. However, randomized-controlled trials on the outcome-modifying effect of metformin in HF are lacking. Interestingly, GLP-1 receptor agonists such as liraglutide have been shown to worsen congestion and to increase cardiovascular adverse events in HFrEF independently from diabetes mellitus type 2 [96,97]. In particular, GLP1 analogs may reduce adipogenesis, improve fat utilization, and induce brown fat differentiation in EAT [98]. Iacobellis et al. showed in a small trial that GLP1A was able to reduce EAT volume, but the study was not designed to detect a difference in hard clinical endpoints [99].

Table 3 summarizes current available therapies targeting epicardial adipose tissue in heart failure.

In the recent future, adipose tissue could be a new target for gene therapy as well. The fibroblast growth factor 21 (FGF21) has been identified as a promising therapeutic agent for type 2 diabetes and other metabolic diseases. The overexpression of the FGF21 in the visceral adipose tissue was associated with an improvement in energy homeostasis. A low-dose intraperitoneal injection of engineered serotype adeno-associated viral vector-FGF21 resulted in reduced whole-body adiposity and inflammatory cytokine release, lower insulin resistance, and glycemic processing [100].

Taken together, these studies raise the question of whether EAT volume alone is the right surrogate marker at all, as the metabolic activity of the EAT may be independent of its total volume. Overall, different interventions seem to lead to similar reductions in EAT in both HFpEF and HFrEF, but we need more clinical trials to investigate the different roles of EAT across the spectrum of LVEF and underlying etiologies.

## 6. Conclusions

Epicardial adipose tissue represents an important determinant for altered cell signals, energetic substrate, and an excessive immune response. Moreover, it shows additional diagnostic and prognostic properties in the HFpEF population, aside from the common markers of inflammation, cardiovascular dysfunction, and fibrosis. On the contrary, EAT is reduced in HFrEF patients, in whom its role is not fully understood and is likely related to a nurturing function. The assessment of EAT in clinical practice could lead to a better understanding of the molecular pathways and biological mechanisms responsible for HF syndromes, where it might represent a promising therapeutic target. In particular, understanding the EAT transcriptional profile in physiological and pathological conditions might represent the keystone to developing disease-modifying therapies in HFpEF, and to deep-phenotyping this not yet fully understood, poly-etiologic clinical syndrome. Until then, EAT remains a risk factor for adverse outcomes and not a treatment target. As effective therapies to reduce the disease burden, particularly of HFpEF, are sparse, EAT may provide novel insights into treatment strategies in the future.

## Figures and Tables

**Table 1:** Studies investigating the role of EAT in heart failure with preserved and mildly reduced ejection fraction.

Manuscript	Study Design	Sample Size	Method	Major Findings
Obokata, 2017 [31]	Retrospective single center study	99 obese HFpEF patients (mean age 65 ± 11 years, 38% male, BMI 40.8 ± 5.6 kg/m^2^), 96 non-obese HFpEF patients (mean age 70 ± 10 years, 38% male, BMI 26.0 ± 2.7 kg/m^2^), 71 non-obese control subjects free of HF (mean age 62 ± 10 years, 42% male, BMI 25.4 ± 2.8 kg/m^2^)	Echocardiography (EAT thickness)	Compared to non-obese HFpEF and controls, obese HFpEF patients had an increased EAT (10 ± 2 versus 7 ± 2 and 6 ± 2 mm; *p* < 0.0001), worse exercise capacity (peak oxygen consumption, 7.7 ± 2.3 versus 10.0 ± 3.4 and 12.9 ± 4.0 mL/min·kg; *p* < 0.0001), increased plasma volume, more concentric LV remodeling, and lower N-proBNP values. Pulmonary capillary wedge pressure was correlated with body mass and plasma volume in obese HFpEF (r = 0.22 and 0.27, both *p* < 0.05) but not in non-obese HFpEF. The increase in heart volumes in obese HFpEF was associated with greater pericardial restraint and heightened ventricular interdependence.
Van Woerden, 2018 [57]	Observational prospective single center study	64 HFpEF patients (median age 70 ± 10.7 years, 63% male, BMI 29.6 ± 5.7 kg/m^2^) vs. 20 controls (median age 66 ± 5.5 years, 65% male, BMI 27.2 ± 4.6 kg/m^2^)	Cardiac magnetic resonance (EAT volume)	Total EAT volume higher in HFpEF compared to controls (107 mL/m^2^ vs. 77 mL/m^2^, *p* < 0.0001). HFpEF patients with atrial fibrillation and/or type 2 diabetes mellitus had more EAT than HF patients without these co-morbidities (116 vs. 100 mL/m^2^, *p* = 0.03, and 120 vs. 97 mL/m^2^, *p* = 0.001, respectively).
Wu, 2020 [51]	National Taiwan University Hospital CMRI registry	163 HFpEF patients (mean age 61 ± 15 years, 63% male, BMI 26 ± 4 kg/ m^2^), 34 HFrEF patients (mean age 55 ± 15 years, 82% male, BMI 25 ± 5 kg/ m^2^), 28 HFmrEF patients, 108 non-HF controls	Cardiac magnetic resonance (EAT volume and intramyocardial mass)	Intramyocardial fat higher in HFpEF than HFrEF patients or non-HF controls [intramyocardial fat content 1.56% (1.26, 1.89) vs. 0.75% (0.50, 0.87) and 1.0% (0.79, 1.15), *p* < 0.05]. Intramyocardial fat correlated with LV diastolic dysfunction parameters in HFpEF patients independently of age, co-morbidities, BMI, gender, and myocardial fibrosis (β = −0.34, *p* = 0.03; β = 0.29, *p* = 0.025; and β = 0.25, *p* = 0.02, respectively).
Gorter, 2020 [54]	Observational prospective single center study	75 HFpEF patients (mean age 74 ± 9 years; 32% male, BMI 29 ± 6 kg/m^2^, 36% obese)	Echocardiography (EAT thickness)	Increased EAT associated with higher right ventricular end-diastolic pressure and with lower VO_2_-max independently from pulmonary venous resistance (OR 1.16 [1.02 to 1.34], *p* = 0.03, and OR 0.64 [0.49 to 0.84], *p* = 0.002, respectively), and obesity (OR 0.69 [0.53 to 0.92], *p* = 0.01). EAT thickness was not associated with left-sided filling pressures.
Koepp, 2020 [60]	Observational prospective single center study	77 patients with HFpEF, obesity and increased EAT (mean age 67 ± 12 years, 32% male, BMI 39.9 ± 6.6 kg/m^2^) vs. 92 patients with HFpEF, obesity and reduced EAT (mean age 66 ± 10 years, 43% male, BMI 34.5 ± 4.2 kg/m^2^)	Echocardiography (EAT thickness)	Obese patients with HFpEF with increased EAT had higher right atrial, pulmonary artery, and pulmonary capillary wedge pressures at rest and during exercise and lower peak oxygen consumption (VO_2_)
Van Woerden, 2021 [55]	Observational prospective single center study	102 HFpEF patients with LVEF > 40% (mean age 70 ± 10 years, male 51%, BMI 29.5 ± 5.8 kg/m^2^)	Cardiac magnetic resonance (EAT volume)	Right ventricular EAT was positively associated with RV mass after adjusting for total EAT, sex, NT-proBNP, renal function, and blood glucose. Atrial EAT was increased in patients with atrial fibrillation compared to those without atrial fibrillation (30 vs. 26 mL/m^2^, *p* = 0.04).
Pugliese, 2021 [56]	Observational prospective single center study	205 HFrEF patients (median age 65 (IQR: 55–74) years, 65% male, BMI 27 (IQR: 21–33) kg/m^2^), 188 HFpEF patients (median age 73 (IQR: 64–80) years, 48% male, BMI 31.5 (IQR: 29–36) kg/m^2^), 44 healthy controls (median age 61 (IQR: 54–70) years, 59% male, BMI 23 (IQR: 22–24) kg/m^2^).	Echocardiography (EAT thickness)	HFpEF patients displayed the highest EAT. In HFpEF, EAT had a direct association with troponin T, C-reactive protein, and right ventriculo–arterial uncoupling, whereas an inverse correlation with peak VO_2_ and AVO2diff was observed. Increased EAT in HFpEF was related to a higher risk of adverse events.
Ying, 2021 [59]	Observational prospective single center study	55 HFpEF patients (mean age 67 ± 11 years, 25% male), 33 controls (mean age 57 ± 10 years, 36% male)	Cardiac magnetic resonance (EAT thickness)	HFpEF patient had higher EAT (4.6 [IQR 2.0]) vs. controls (3.2 [IQR 1.4], *p* < 0.001). Increased EAT was associated with lower well-being scores.
Van Woerden, 2022 [58]	Observational prospective single center study	105 HFpEF patients (mean age 72 ± 8 years, 50% male, and mean LVEF 53 ± 8%), median follow-up of 24 (17–25) months	Cardiac magnetic resonance (EAT volume)	EAT was associated with all-cause mortality (HR, 2.06 [1.26–3.37], *p* = 0.004) and HF hospitalizations (HR, 1.54 [1.04–2.30], *p* = 0.03).
Venkateshvaran, 2022 [45]	Prospective, multinational study (PROMIS-HFpEF)	182 HFpEF patients: n = 54 patients with increased EAT ≥ 9 mm (mean age 73 (68–77) years, 57% male, and mean LVEF 62 (56–66)%), vs. n = 128 patients with reduced EAT < 9 mm (mean age 76 (70–82) years, 54% male, mean LVEF 58 (54–64)%).	Echocardiography (EAT thickness)	Patients with increased EAT had higher body mass index (32 (28–40) vs. 27 (23–30) kg/m^2^; *p* < 0.001), lower NT-proBNP (466 (193–1133) vs.1120 (494–1990) pg/mL; *p* < 0.001), smaller indexed LV end-diastolic and LA volumes. EAT was moderately and significantly correlated with BMI (r = 0.49, *p* < 0.001). When adjusted for BMI, EAT was associated with LV septal wall thickness (B = 1.02, [1–1.04], *p* = 0.018) and mitral E wave deceleration time (B01.03, [1.01–1.05], *p* = 0.005). Increased EAT was associated with proteomic markers of adipose biology and inflammation, insulin resistance, endothelial dysfunction, and dyslipidaemia.
Jin, 2022 [61]	Observational retrospective, 2 different cohorts	99 HFpEF patients (mean age 65 ± 11 years, 63% male, BMI 29 ± 6.3 kg/m^2^); 366 HFrEF/HFmrEF patients (mean age 57 ± 11 years, 84% male, BMI 27 ± 5.6 kg/m^2^); 149 controls (mean age 58 ± 10.8 years, 46% male, BMI 25 ± 3.9 kg/m^2^).	Echocardiography (EAT thickness)	EAT thickness lower in HFrEF/HFmrEF (7.3 ± 2.5) vs. HFpEF (8.3 ± 2.6 mm, *p* < 0.05). Greater EAT thickness associated with higher LV and LA function in HFrEF but not in HFpEF. Increased EAT associated with LA dysfunction in HFpEF but not in HFrEF/HFmrEF.

**Table 2:** Studies investigating the role of EAT in heart failure with reduced ejection fraction.

Manuscript	Study Design	Sample Size	Method	Major Findings
Doesch, 2010 [36]	Retrospective single center study	66 patients with symptomatic HF and LVEF ≤ 35% (mean age 63 ± 2 years, 82% male, BMI 27 ± 4 kg/m^2^), 32 controls (mean age 57 ± 11 years, 78% male, BMI 28 ± 4 kg/m^2^)	Cardiac magnetic resonance (EAT volume)	Reduced EAT volume and mass in HfrEF irrespective of underlying aetiology. Lower EAT mass/LV mass ratio compared to healthy controls.
Tromp, 2021 [71]	Observational prospective nationwide study (Canada)	204 patients with HF diagnosis (mean age 55 ± 11 years, 82% male, BMI 26 kg/m^2^), 113 community-based controls without HF (mean age 59 ± 10 years, 44% male, BMI 24 kg/m^2^)	Cardiac magnetic resonance (EAT volume) and echocardiography (EAT thickness)	EAT mass higher in HfrEF
Pugliese, 2021 [56]	Observational prospective single center study	205 HfrEF patients (median age 65 (IQR: 55–74) years, 65% male, BMI 27 (IQR: 21–33) kg/m^2^), 188 HfpEF patients (median age 73 (IQR: 64–80) years, 48% male, BMI 31.5 (IQR: 29–36) kg/m^2^), 44 healthy controls (median age 61 (IQR: 54–70) years, 59% male, BMI 23 (IQR: 22–24) kg/m^2^).	Echocardiography (EAT thickness)	Reduced EAT thickness in HfrEF as compared to HfpEF and healthy controls. In HfrEF, a reduced EAT thickness was associated with higher NT-proBNP, hs-CRP, and hs-TnT values; with a reduced execise capacity as expressed by peak VO_2_; and with an increased LV mass. Worse cardiovascular outcome in HFrEF patients with reduced EAT thickness.

**Table 3:** Future perspectives on drugs targeting EAT.

Treatment with Potential Effect on EAT	Rationale	Evidence
Statins	Pleiotropic effect (especially on myocardial inflammation)	Reduction in EAT-secreted inflammatory mediators [72] Improvement in cardiac remodeling in rats [71]
Anticytokines (e.g., anti-TNF alpha, anti-IL1)	Reduction in systemic inflammation	Indirect (and controversial) evidence on reduction in systemic inflammatory biomarkers with hypothesis-generating data [74,75,76,77,78,79]
SGLT2 inhibitors	Reduction in cardiac inflammation and fibrosis	Reduction in EAT mass and fibrosis and inflammatory biomarkers [85,86]
Other oral antidiabetic agents (e.g., metformin, GLP1-RA)	Anti-inflammatory and anti-oxidative stress effects, role on adipogenesis and adipocyte function	Reduction in EAT mass and EAT-related inflammation with hypothesis generated through controversial data
Fibroblast growth factor 21 gene therapy	Improvement in energy homeostasis in visceral adipose tissue	Intraperitoneal injection associated with lower adiposity, inflammatory cytokines, insulin resistance, and glycemic processing in mice [89]

EAT: epicardial adipose tissue; SGLT2: sodium glucose transporter 2; GLP1-RA: glucose lowering protein 1 receptor agonist.

## Data Availability

Not applicable.
